# Supplementary material for: Frequent FOXA1-Activating Mutations in Extramammary Paget’s Disease
Source: Cancers (Basel). 2020 Mar 29;12(4):820. doi: 10.3390/cancers12040820 (PMC7226542; doi:10.3390/cancers12040820)
Supplement: Supplementary file 1 [file cancers-12-00820-s001.pdf]

Supplementary Information

Supplementary Figures pp. 2–4

Figure S1. Validation of the genomic breakpoints involving *GAS6* and *FOXA1* in UPN1

Figure S2. Validation of the *FOXA1* g.38064406G>A mutation identified in UPN2

Figure S3. Additional gene set enrichment analyses

Supplementary Tables pp. 5–31

Table S1. Detailed clinical characteristics of each patient

Table S2. Genes and regions analyzed by targeted sequencing

Table S3. Nonsynonymous mutations identified by whole-genome sequencing

Table S4. Nonsynonymous mutations identified by whole-exome sequencing

Table S5. Summary of possible driver mutations identified in this study

Table S6. Top 200 differentially expressed genes

Supplementary Figures

Figure S1. Validation of the genomic breakpoints involving *GAS6* and *FOXA1* in UPN1

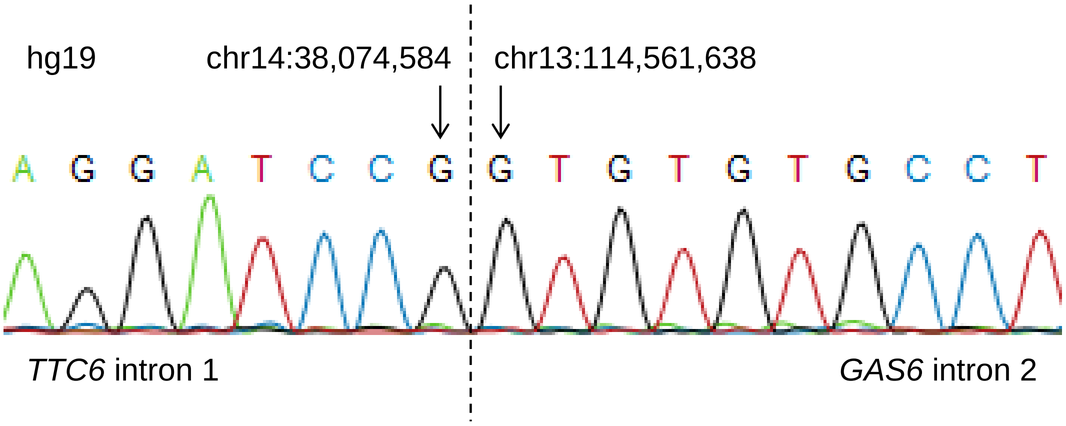

Sanger sequencing validating the structural variation identified in UPN1. The hg19 genome coordinate is used to represent the positions of the breakpoint.

Figure S2. Validation of the *FOXA1* g.38064406G>A mutation identified in UPN2

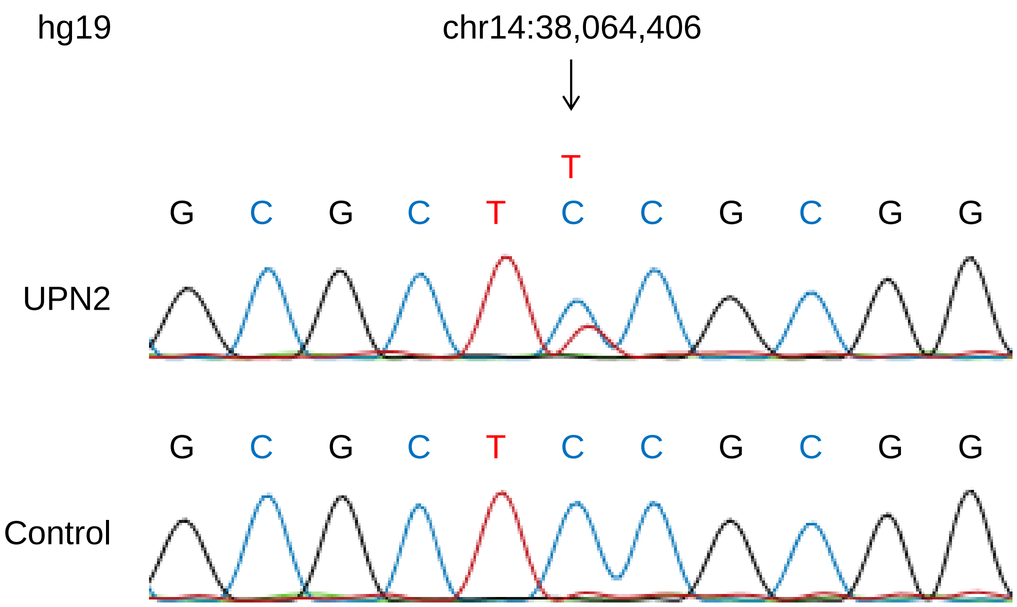

Sanger sequencing validating the presence of C>T (G>A) nucleotide alteration at 38,064,406 on chromosome 14.

Figure S3. Additional gene set enrichment analyses

A

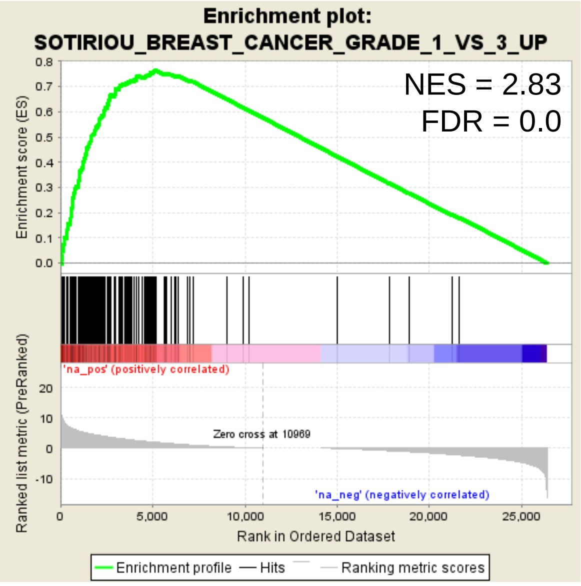

B

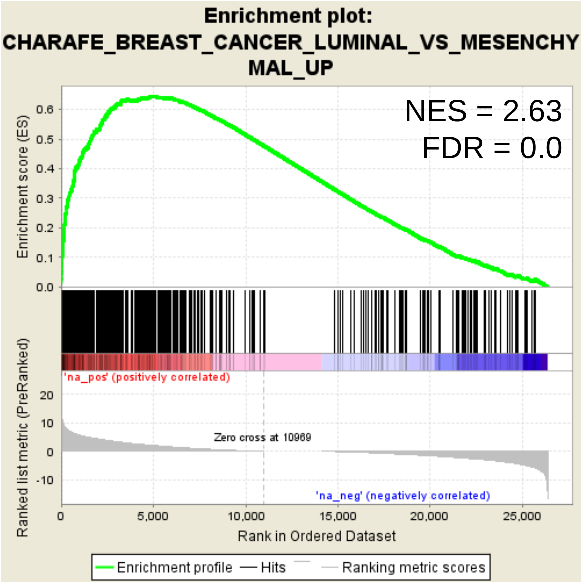

Enrichment plots showing the upregulation of genes associated with (A) high-grade breast cancer (SOTIRIOU_BREAST_CANCER_GRADE_1_VS_3_UP, the most significantly enriched in the C2 gene set database) and (B) luminal breast cancer (CHARAFE_BREAST_CANCER_LUMINAL_VS_MESENCHYMAL_UP, the fourth most significantly enriched). NES, normalized enrichment score; FDR, false discovery rate.

Table S1. Detailed clinical characteristics of each patient

UPN	Age / gender	Age at onset	Primary location	Dermal invasion	Metastasis of SLN	Frozen / FFPE	Analysis	FOXA1 expression	ER expression
1	55 / F	53	Perineal	*In situ*	-	Frozen	WGS/RNA/TGS	+	+
2	70 / M	68	Penoscrotal	Invasive	-	Frozen	WGS/RNA/TGS	+	+
3	73 / F	72	Perineal	*In situ*	-	Frozen	RNA/TGS	+	+
4	65 / F	64	Perineal	Invasive	-	Frozen	TGS	+	+
5	89 / M	88	Penoscrotal	Invasive	+	Frozen	RNA/TGS	+	+
6	76 / F	75	Perineal	Invasive	-	Frozen	RNA/TGS	+	+
7	71 / F	68	Perineal	*In situ*	-	Frozen	RNA/TGS	+	+
8	79 / M	78	Penoscrotal	Invasive	-	Frozen	RNA/TGS	+	+
9	58 / M	53	Penoscrotal	Invasive	-	Frozen	RNA/TGS	+	NA
10	66 / M	65	Penoscrotal	*In situ*	-	Frozen	TGS	+	+
11	72 / M	67	Penoscrotal	Invasive	+	FFPE	WES/TGS	+	+
12	80 / M	79	Penoscrotal	*In situ*	-	FFPE	WES/TGS	+	+
13	60 / M	59	Penoscrotal	Invasive	+	FFPE	WES/TGS	+	+
14	67 / M	64	Inguinal	Invasive	+	FFPE	WES/TGS	+	-
15	70 / M	70	Perineal	Invasive	-	FFPE	WES/TGS	+	-
16	73 / F	70	Perineal	*In situ*	-	FFPE	WES/TGS	+	-
17	88 / F	84	Perineal	*In situ*	-	FFPE	WES/TGS	+	+
18	80 / F	79	Perineal	*In situ*	-	FFPE	WES/TGS	+	-
19	82 / F	79	Perineal	Invasive	-	FFPE	WES/TGS	+	-
20	72 / M	69	Perineal	Invasive	-	FFPE	WES/TGS	+	-
21	69 / F	65	Perineal	*In situ*	-	FFPE	WES/TGS	+	+
22	78 / M	75	Penoscrotal	Invasive	-	FFPE	TGS	+	+
23	82 / M	81	Penoscrotal	Invasive	-	FFPE	TGS	+	++
24	90 / F	83	Perineal	*In situ*	NA	FFPE	TGS	+	+
25	65 / M	57	Penoscrotal	*In situ*	NA	FFPE	TGS	+	+
26	76 / M	76	Penoscrotal	*In situ*	NA	FFPE	TGS	+	-
27	64 / F	58	Perineal	*In situ*	-	FFPE	TGS	+	++
28	62 / M	59	Penoscrotal	Invasive	-	FFPE	TGS	+	+
29	85 / M	84	Penoscrotal	Invasive	NA	FFPE	TGS	+	-
30	74 / F	66	Axillary	*In situ*	NA	FFPE	TGS	+	+
31	73 / M	68	Penoscrotal	*In situ*	NA	FFPE	TGS	+	-
32	81 / M	80	Penoscrotal	Invasive	-	FFPE	TGS	+	-
33	71 / M	61	Penoscrotal	Invasive	NA	FFPE	TGS	+	+
34	78 / M	77	Penoscrotal	Invasive	NA	FFPE	TGS	+	++
35	92 / F	NA	Perineal	Invasive	NA	FFPE	TGS	+	+
36	87 / F	NA	Axillary	*In situ*	NA	FFPE	TGS	+	++
37	64 / M	61	Penoscrotal	*In situ*	NA	FFPE	TGS	+	++
38	83 / M	NA	Penoscrotal	*In situ*	NA	FFPE	TGS	+	+

39	77/M	77	Penoscrotal	Invasive	-	Frozen	WES/TGS	NA	NA
40	57/F	54	Vulval	*In situ*	-	Frozen	WES/TGS	+	-
41	78/F	78	Perianal	*In situ*	-	Frozen	WES/TGS	+	-
42	77/M	76	Perianal	*In situ*	-	Frozen	WES/TGS	+	+
43	59/M	53	Penoscrotal	Invasive	-	Frozen	WES/TGS	+	-
44	68/F	67	Perianal	*In situ*	-	Frozen	WES/TGS	+	-
45	69/M	64	Mons pubis	Invasive	-	Frozen	WES/TGS	+	-
46	75/M	74	Inguinal	Invasive	+	Frozen	WES/TGS	+	+
47	74/M	74	Penoscrotal	*In situ*	-	Frozen	WES/TGS	+	+
48	71/M	71	Inguinal	*In situ*	-	Frozen	WES/TGS	+	-

SLN, sentinel lymph node: FFPE, formalin-fixed paraffin-embedded; ER, estrogen receptor; WGS, whole-genome sequencing; RNA, RNA sequencing; TGS, targeted gene sequencing; WES, whole-exome sequencing; NA, not available

Table S2. Genes and regions analyzed by targeted sequencing

Genes (coding regions + 25 bp targeted)							
ABCB1	*ABCC2*	*ABL1*	*ABL2*	*ACO1*	*ACOX1*	*ACRC*	*ACTG1*
ACVR1B	*ADAM28*	*ADGRB3*	*ADIPOR1*	*AGAP2*	*AHNAK*	*AIM1*	*AK2*
AKT1	*AKT2*	*AKT3*	*ALDH1B1*	*ALK*	*ALPK2*	*ANK3*	*ANTXR1*
APC	*AQP7*	*ARHGEF16*	*ARID1A*	*ARID2*	*ARMCX4*	*ASXL1*	*ATM*
ATN1	*ATP1A1*	*ATP6V0C*	*ATP8A1*	*ATP8B2*	*ATRX*	*BAHD1*	*BCL11A*
BCLAF1	*BRAF*	*BRCA1*	*BRCA2*	*BRD4*	*C11orf30*	*C11orf80*	*C14orf159*
C16orf82	*C16orf89*	*C1orf127*	*C1QL1*	*C8orf44*	*CACNA1C*	*CAMTA1*	*CASC5*
CASP8	*CAST*	*CBFB*	*CBL*	*CCDC116*	*CCDC148*	*CCDC40*	*CDA*
CDC42BPA	*CDH1*	*CDK20*	*CDK4*	*CDKAL1*	*CDKN1B*	*CDKN2A*	*CDKN2B*
CEBPA	*CFAP221*	*CHD2*	*CHD7*	*CHGB*	*CHIC2*	*CHRM5*	*CLCN3*
CLPS	*CNNM4*	*COBLL1*	*CREBBP*	*CRLF2*	*CROT*	*CRY1*	*CRY2*
CRYBA4	*CSF1R*	*CTCF*	*CTNNB1*	*CTNND2*	*CUL4B*	*CYB561D1*	*CYGB*
CYP19A1	*CYP26B1*	*CYP27B1*	*CYP2A6*	*CYP2B6*	*CYP2C19*	*CYP2C9*	*CYP2D6*
CYP2E1	*DCAF10*	*DDR1*	*DDR2*	*DDX3X*	*DDX53*	*DGKD*	*DHPS*
DHX32	*DHX38*	*DLC1*	*DNMT1*	*DNMT3A*	*DPCR1*	*DPYD*	*DST*
DUSP14	*DUSP4*	*DYNAP*	*EGFL6*	*EGFR*	*ELOVL2*	*EP300*	*EPHA7*
EPPK1	*ERBB2*	*ERBB3*	*ERBB4*	*ERG*	*ERP29*	*ERRFI1*	*ESR1*
ESR2	*EYA1*	*EZH2*	*FAM208A*	*FANCD2*	*FAT1*	*FBXW7*	*FDXACB1*
FGFR1	*FGFR2*	*FGFR3*	*FGFR4*	*FITM1*	*FLG*	*FLNB*	*FLT1*
FLT3	*FLT4*	*FRMD7*	*FRYL*	*FSTL5*	*FUT7*	*GATA3*	*GLIS3*
GLUD1	*GNA11*	*GNAQ*	*GNAS*	*GNE*	*GNPTAB*	*GPC5*	*GPR116*
GPR179	*GPRC6A*	*GRHL2*	*GSTP1*	*GTPBP4*	*H3F3A*	*H3F3B*	*HDAC9*
HECTD4	*HIST1H1A*	*HIST1H1B*	*HIST1H1C*	*HIST1H1D*	*HIST1H1E*	*HIST1H1T*	*HIST1H2AA*
HIST1H2AB	*HIST1H2AC*	*HIST1H2AD*	*HIST1H2AE*	*HIST1H2AG*	*HIST1H2AH*	*HIST1H2AI*	*HIST1H2AJ*
HIST1H2AK	*HIST1H2AL*	*HIST1H2AM*	*HIST1H2BA*	*HIST1H2BB*	*HIST1H2BC*	*HIST1H2BD*	*HIST1H2BE*
HIST1H2BF	*HIST1H2BG*	*HIST1H2BH*	*HIST1H2BI*	*HIST1H2BJ*	*HIST1H2BK*	*HIST1H2BL*	*HIST1H2BM*
HIST1H2BN	*HIST1H2BO*	*HIST1H3A*	*HIST1H3B*	*HIST1H3C*	*HIST1H3D*	*HIST1H3E*	*HIST1H3F*
HIST1H3G	*HIST1H3H*	*HIST1H3I*	*HIST1H3J*	*HIST1H4A*	*HIST1H4B*	*HIST1H4C*	*HIST1H4D*
HIST1H4E	*HIST1H4F*	*HIST1H4G*	*HIST1H4H*	*HIST1H4I*	*HIST1H4J*	*HIST1H4K*	*HIST1H4L*
HIST2H2AA3	*HIST2H2AA4*	*HIST2H2AB*	*HIST2H2AC*	*HIST2H2BE*	*HIST2H2BF*	*HIST2H3A*	*HIST2H3C*
HIST2H3D	*HIST2H4A*	*HIST2H4B*	*HIST3H2A*	*HIST3H2BB*	*HIST3H3*	*HIST4H4*	*HLA-B*
HMCN1	*HNF1A*	*HOXA3*	*HPS5*	*HRAS*	*HRNR*	*HSPG2*	*IDH1*
IDH2	*IDI1*	*IGSF10*	*IGSF22*	*IKZF1*	*IL1RN*	*IL2RA*	*IL2RB*
IL2RG	*IL6ST*	*ILKAP*	*INO80D*	*INPP4B*	*IQSEC1*	*ITGAV*	*JAG2*
JAK1	*JAK2*	*JAK3*	*JARID2*	*JMJD6*	*KALRN*	*KANK2*	*KDM1A*
KDM6A	*KDM7A*	*KDR*	*KIAA0391*	*KIT*	*KMT2A*	*KMT2C*	*KNG1*
KRAS	*KRTAP10-2*	*KRTAP4-11*	*KSR2*	*LAMA2*	*LAMC1*	*LCK*	*LCLAT1*
LCTL	*LILRB5*	*LMTK3*	*LOXL4*	*LRCH2*	*LRP6*	*LRR1*	*LTK*
MACF1	*MAP2K1*	*MAP2K2*	*MAP2K4*	*MAP3K1*	*MAPK1*	*MED13*	*MED23*

MED24	*MET*	*MGA*	*MGAM2*	*MLH1*	*MOV10L1*	*MPL*	*MRTO4*
MST1R	*MTOR*	*MTR*	*MUC4*	*MXRA5*	*MYB*	*MYC*	*MYD88*
MYOCD	*MYRF*	*NACA*	*NACC2*	*NADK*	*NBPF10*	*NCOR1*	*NEFH*
NELL2	*NF1*	*NIM1K*	*NLRP8*	*NME2*	*NOTCH1*	*NOTCH2*	*NPIPA5*
NPIPB15	*NPIPB5*	*NPM1*	*NRAS*	*OGDH*	*OPCML*	*OR4A16*	*OR52N1*
PABPC1	*PAGE1*	*PANX2*	*PCDHA9*	*PCDHB4*	*PCNX*	*PCSK1*	*PCSK6*
PDGFRA	*PDGFRB*	*PDK1*	*PDZD8*	*PHF6*	*PIGN*	*PIK3C2A*	*PIK3CA*
PIK3R1	*PITPNC1*	*PLA2G6*	*PLCL1*	*PLIN4*	*PLXDC2*	*PLXNA4*	*POTEG*
PPFIBP1	*PPP6C*	*PRUNE2*	*PSMB1*	*PSMB2*	*PSMB5*	*PSMD1*	*PSMD2*
PTCH1	*PTEN*	*PTGFRN*	*PTPN11*	*PTPRF*	*RAB40A*	*RAC1*	*RAF1*
RARA	*RARB*	*RARG*	*RB1*	*RET*	*RNASE1*	*RNF43*	*ROCK2*
ROR1	*ROS1*	*RPS6KB1*	*RRP9*	*RUNX1*	*RXRA*	*RXRB*	*RXRG*
SACS	*SCN2A*	*SCP2*	*SCRT1*	*SDHB*	*SEC31A*	*SERPINB3*	*SETDB1*
SF3B1	*SHH*	*SHOC2*	*SLC12A8*	*SLC22A1*	*SLC22A2*	*SLC2A1*	*SLC31A1*
SLC34A2	*SLC44A4*	*SLC45A3*	*SLC4A1AP*	*SLC4A7*	*SLC6A1*	*SLCO1B1*	*SLCO1B3*
SMAD4	*SMARCA4*	*SMARCB1*	*SMC5*	*SMG1*	*SMO*	*SMPD4*	*SNCAIP*
SNX31	*SOD2*	*SOGA1*	*SOS1*	*SPDYE1*	*SPEN*	*SPG11*	*SPRED1*
SPTAN1	*SRC*	*STAG2*	*STARD13*	*STK11*	*STK19*	*SUFU*	*SYNE1*
TANC1	*TAS2R38*	*TBL1XR1*	*TBX3*	*TBXA2R*	*TCEB3C*	*TCEB3CL*	*TEKT4*
TET2	*TIAL1*	*TM9SF2*	*TMEM51*	*TNFRSF25*	*TP53*	*TRIO*	*TRPV4*
TRRAP	*TSHZ3*	*TTC22*	*TUBA3E*	*TXNRD1*	*TYK2*	*UBE3B*	*UBE4A*
UBTD2	*UGT1A1*	*UGT2B11*	*UNC13A*	*UQCRFS1*	*USP19*	*USP4*	*USP6NL*
VHL	*VPS41*	*WASF3*	*WHSC1*	*WT1*	*WWP1*	*XIRP2*	*YES1*
YIPF1	*ZC3H7A*	*ZKSCAN2*	*ZMYM3*	*ZMYM4*	*ZNF141*	*ZNF195*	*ZNF208*
ZNF217	*ZNF236*	*ZNF285*	*ZNF429*	*ZNF479*	*ZNF528*	*ZNF534*	*ZNF587B*
ZNF611	*ZNF676*	*ZNF727*	*ZNF728*	*ZNF729*	*ZNF735*	*ZNF750*	*ZNF765*
ZNF816	*ZNF85*	*ZSWIM8*					
Regions							
Whole UTR/exon/intron of *GAS6* (chr13:113819854-113864930 @ hg38, 44 kb)							
Whole UTR/exon/intron of *FOXA1* and 200-kb upstream (chr14:37585355-37843301 @ hg38, 256 kb)							

UTR, untranslated region

Table S3. Nonsynonymous mutations identified by whole-genome sequencing

UPN	Gene	Reference	Nucleic acid change	Amino acid change	VAF	ClinVar	COSMIC
1	*ADAM11*	NM_002390	c.826-2A>C	(exon 11)	0.12	-	-
1	*AHNAK2*	NM_138420	c.6181G>C	p.V2061L	0.16	-	-
1	*BEAN1*	NM_001178020	c.244_245insTTTTTTAATG	p.H82Lfs*3	0.10	-	-
1	*CALML6*	NM_138705	c.433G>A	p.E145K	0.10	-	-
1	*CCDC141*	NM_173648	c.4018G>T	p.G1340C	0.10	-	-
1	*CNOT1*	NM_206999	c.4635A>T	p.L1545F	0.10	-	-
1	*CORIN*	NM_006587	c.618-3T>-	(exon 5)	0.11	-	-
1	*CORO2B*	NM_006091	c.881A>G	p.N294S	0.11	-	-
1	*EMX1*	NM_004097	c.241A>C	p.T81P	0.11	-	-
1	*F10*	NM_000504	c.626A>C	p.D209A	0.15	-	-
1	*FAM47C*	NM_001013736	c.1373G>A	p.R458Q	0.10	-	-
1	*G2E3*	NM_017769	c.1393G>T	p.G465C	0.11	-	-
1	*IQSEC2*	NM_001111125	c.3574T>G	p.Y1192D	0.18	-	-
1	*LLGL2*	NM_004524	c.1732C>T	p.Q578*	0.12	-	-
1	*LOC100129520*	NM_001195272	c.1148C>T	p.P383L	0.12	-	-
1	*MDK*	NM_002391	c.406+2T>G	(exon 4)	0.16	-	-
1	*MUC2*	NM_002457	c.4370C>T	p.P1457L	0.08	-	-
1	*MYBPC1*	NM_206820	c.2290G>T	p.A764S	0.07	-	-
1	*MYCBP2*	NM_015057	c.2434A>C	p.K812Q	0.16	-	-
1	*NFATC3*	NM_004555	c.91T>G	p.S31A	0.14	-	-
1	*NOS2*	NM_000625	c.2081T>C	p.I694T	0.10	-	-
1	*NRAS*	NM_002524	c.430A>C	p.T144P	0.10	-	-
1	*NUMB*	NM_003744	c.625G>A	p.E209K	0.12	-	-
1	*OBSCN*	NM_052843	c.11536T>C	p.Y3846H	0.09	-	-
1	*OR1L4*	NM_001005235	c.143T>C	p.L48P	0.11	-	-
1	*PAMR1*	NM_015430	c.509G>C	p.S170T	0.07	-	-
1	*PCM1*	NM_006197	c.1471C>A	p.Q491K	0.08	-	-
1	*PDSS2*	NM_020381	c.94A>C	p.I32L	0.13	-	-
1	*PLCL1*	NM_006226	c.2161C>G	p.L721V	0.11	-	-
1	*PRAMEF1*	NM_023013	c.41G>T	p.G14V	0.11	-	-
1	*PRAMEF6*	NM_001010889	c.1211T>G	p.I404R	0.15	-	-
1	*PRAMEF6*	NM_001010889	c.1222T>A	p.L408I	0.17	-	-
1	*PTPRH*	NM_002842	c.2227C>T	p.H743Y	0.14	-	-
1	*PTPRQ*	NM_001145026	c.3368G>A	p.G1123E	0.09	-	-
1	*RIMS1*	NM_014989	c.2054T>C	p.V685A	0.13	-	-
1	*SEPT8*	NM_015146	c.528C>G	p.D176E	0.10	-	-
1	*SOX8*	NM_014587	c.982C>A	p.P328T	0.15	-	-
1	*STXBP5*	NM_001127715	c.2205G>T	p.K735N	0.10	-	-
1	*TMCO4*	NM_181719	c.856T>G	p.W286G	0.12	-	-

1	*TRIM43*	NM_138800	c.1188_1189delAG	p.K398Tfs*10	0.14	-	-
1	*ZNF517*	NM_213605	c.1094A>C	p.D365A	0.11	-	-
2	*ABCA10*	NM_080282	c.2456C>T	p.P819L	0.18	-	-
2	*ADAMTS10*	NM_030957	c.457G>A	p.E153K	0.19	-	-
2	*APOE*	NM_000041	c.371C>T	p.A124V	0.29	-	-
2	*APOL5*	NM_030642	c.1141C>T	p.L381F	0.23	-	-
2	*C10orf90*	NM_001004298	c.193G>C	p.E65Q	0.20	-	-
2	*C14orf105*	NM_001283056	c.653-4T>-	(exon 6)	0.19	-	-
2	*CCDC168*	NM_001146197	c.8479G>C	p.E2827Q	0.20	-	-
2	*CCT6A*	NM_001762	c.1065+1G>C	(exon 9)	0.22	-	-
2	*CD300C*	NM_006678	c.494G>C	p.R165T	0.22	-	-
2	*CFAP54*	NM_001306084	c.387G>C	p.K129N	0.36	-	-
2	*CRYBG3*	NM_153605	c.5167G>A	p.E1723K	0.36	-	-
2	*DST*	NM_001144769	c.170C>T	p.S57L	0.15	-	-
2	*EQTN*	NM_020641	c.280C>G	p.L94V	0.41	-	-
2	*FAM129B*	NM_022833	c.587A>G	p.N196S	0.16	-	-
2	*FASN*	NM_004104	c.1771G>A	p.D591N	0.26	-	-
2	*FASTK*	NM_006712	c.1039+4C>G	(exon 5)	0.23	-	-
2	*FCAR*	NM_002000	c.460G>C	p.D154H	0.22	-	-
2	*FOXA1*	NM_001310135	g.38064406G>A	-	0.20	-	-
2	*FREM3*	NM_001168235	c.4063C>A	p.L1355I	0.25	-	-
2	*FUT9*	NM_006581	c.68T>C	p.M23T	0.15	-	-
2	*GGT1*	NM_013421	c.1673C>T	p.S558L	0.13	-	-
2	*GLDN*	NM_181789	c.295G>A	p.E99K	0.28	-	-
2	*GUCY1B3*	NM_000857	c.565G>C	p.E189Q	0.25	-	-
2	*HDGFRP3*	NM_016073	c.607-5T>-	(exon 6)	0.23	-	-
2	*HIST1H2BC*	NM_003526	c.72G>C	p.K24N	0.25	-	-
2	*HORMAD1*	NM_032132	c.872-5T>-	(exon 12)	0.34	-	-
2	*INTS7*	NM_015434	c.1816-5->T	(exon 14)	0.21	-	-
2	*LAMA2*	NM_000426	c.6307G>C	p.E2103Q	0.17	-	-
2	*MOCS1*	NM_005943	c.250+3G>A	(exon 1)	0.24	-	-
2	*MUC12*	NM_001164462	c.994C>A	p.P332T	0.20	-	-
2	*MUM1*	NM_032853	c.2020G>A	p.E674K	0.20	-	-
2	*MYPOP*	NM_001012643	c.337G>C	p.E113Q	0.22	-	-
2	*NDUFS5*	NM_004552	c.70G>A	p.E24K	0.25	-	-
2	*NFATC4*	NM_001288802	c.61G>C	p.E21Q	0.18	-	-
2	*NMRK2*	NM_170678	c.436G>A	p.D146N	0.24	-	-
2	*NOTCH1*	NM_017617	c.1904-3C>A	(exon 12)	0.18	-	-
2	*NR4A3*	NM_006981	c.1299T>G	p.H433Q	0.22	-	-
2	*NRIP1*	NM_003489	c.2650C>A	p.H884N	0.33	-	-
2	*PEAR1*	NM_001080471	c.133C>T	p.R45C	0.16	-	-
2	*PIK3CA*	NM_006218	c.241G>A	p.E81K	0.21	-	COSM271871

2	*POM121*	NM_172020	c.822delG	p.K275Rfs*59	0.23	-	-
2	*PRUNE2*	NM_015225	c.9051-3T>-	(exon 16)	0.16	-	-
2	*RGPD3*	NM_001144013	c.2107G>A	p.E703K	0.30	-	-
2	*RPTOR*	NM_020761	c.3670G>A	p.D1224N	0.16	-	-
2	*SALL1*	NM_002968	c.1763dupC	p.G589Rfs*6	0.26	-	-
2	*SLC18A3*	NM_003055	c.770_772delTGT	p.L257del	0.20	-	-
2	*SPATA13*	NM_001166271	c.755G>A	p.S252N	0.25	-	-
2	*SRL*	NM_001098814	c.530A>C	p.E177A	0.36	-	-
2	*SRSF10*	NM_054016	c.749G>A	p.R250K	0.17	-	-
2	*TAP1*	NM_000593	c.1039C>T	p.R347W	0.31	-	-
2	*THSD7A*	NM_015204	c.1630C>T	p.R544C	0.27	-	-
2	*TMEM182*	NM_144632	c.94C>G	p.L32V	0.23	-	-
2	*TMPRSS13*	NM_001077263	c.1136G>A	p.W379*	0.18	-	-
2	*TNK2*	NM_005781	c.2121C>G	p.F707L	0.22	-	-
2	*TP53BP2*	NM_005426	c.1973C>G	p.S658*	0.24	-	-
2	*TRIOBP*	NM_001039141	c.31G>A	p.E11K	0.21	-	-
2	*UBR1*	NM_174916	c.463G>C	p.E155Q	0.25	-	-
2	*USE1*	NM_018467	c.232-1G>C	(exon 4)	0.17	-	-
2	*ZBED5*	NM_021211	c.116T>C	p.L39S	0.17	-	-
2	*ZNF286B*	NM_001145045	c.1540C>G	p.Q514E	0.15	-	-
2	*ZNF292*	NM_015021	c.266G>A	p.R89Q	0.24	-	-

VAF, variant allele frequency; COSMIC, Catalogue Of Somatic Mutations In Cancer

Table S4. Nonsynonymous mutations identified by whole-exome sequencing

UPN	Gene	Reference	Nucleic acid change	Amino acid change	VAF	ClinVar	COSMIC
12	*CNNM4*	NM_020184	c.727C>T	p.L243F	0.14	-	-
12	*EPPK1*	NM_031308	c.2521T>C	p.Y841H	0.07	-	-
12	*ILKAP*	NM_030768	c.714+1G>A	(exon 8)	0.21	-	-
12	*INO80D*	NM_017759	c.1638delA	p.K546Nfs*49	0.14	-	-
12	*PANX2*	NM_052839	c.508G>A	p.E170K	0.06	-	-
12	*STARD13*	NM_052851	c.2032G>A	p.V678I	0.07	-	-
12	*TNFRSF25*	NM_003790	c.998C>T	p.P333L	0.2	-	-
12	*TUBA3E*	NM_207312	c.322T>C	p.Y108H	0.05	-	-
13	*KRTAP10-2*	NM_198693	c.233C>T	p.S78L	0.18	-	-
13	*POTEG*	NM_001005356	c.35C>T	p.S12F	0.14	-	-
14	*ARHGEF16*	NM_014448	c.544G>A	p.E182K	0.14	-	-
14	*ARMCX4*	NM_001256155	c.3155C>A	p.S1052Y	0.37	-	-
14	*ATN1*	NM_001940	c.496C>A	p.P166T	0.13	-	-
14	*ATN1*	NM_001940	c.2558C>G	p.S853C	0.24	-	-
14	*BCL11A*	NM_018014	c.914C>T	p.P305L	0.18	-	-
14	*C16orf82*	NM_001145545	c.43G>C	p.E15Q	0.24	-	-
14	*C1orf127*	NM_001170754	c.1924G>A	p.D642N	0.13	-	-
14	*C1QL1*	NM_006688	c.478G>A	p.D160N	0.26	-	-
14	*CAMTA1*	NM_015215	c.1636G>C	p.E546Q	0.26	-	-
14	*CHD2*	NM_001271	c.3004G>T	p.A1002S	0.17	-	COSM3706827
14	*CHRM5*	NM_012125	c.527G>A	p.R176Q	0.17	-	-
14	*COBLL1*	NM_014900	c.2462C>G	p.S821C	0.13	-	-
14	*CRYBA4*	NM_001886	c.74G>A	p.R25Q	0.17	-	-
14	*CYP27B1*	NM_000785	c.40C>T	p.R14C	0.25	-	-
14	*CYP2E1*	NM_000773	c.1478C>T	p.S493L	0.2	-	-
14	*DCAF10*	NM_024345	c.1172C>T	p.S391L	0.25	-	-
14	*DLC1*	NM_006094	c.2028C>A	p.F676L	0.44	-	-
14	*ELOVL2*	NM_017770	c.445C>T	p.H149Y	0.2	-	-
14	*EPHA7*	NM_004440	c.962C>A	p.S321Y	0.15	-	-
14	*EPPK1*	NM_031308	c.5620C>T	p.R1874C	0.34	-	-
14	*ERP29*	NM_006817	c.579G>C	p.E193D	0.17	-	-
14	*EYA1*	NM_000503	c.1318C>T	p.R440W	0.16	-	-
14	*FITM1*	NM_203402	c.196delT	p.F66Lfs*24	0.2	-	-

14	*FLG*	NM_002016	c.9896G>C	p.G3299A	0.18	-	-
14	*GLUD1*	NM_005271	c.283G>A	p.E95K	0.28	-	-
14	*GPC5*	NM_004466	c.232C>A	p.Q78K	0.23	-	-
14	*GPRC6A*	NM_148963	c.2393T>G	p.F798C	0.13	-	-
14	*HIST1H2BB*	NM_021062	c.72G>C	p.K24N	0.18	-	COSM1169071
14	*HPS5*	NM_007216	c.244C>G	p.R82G	0.19	-	-
14	*HSPG2*	NM_005529	c.9352C>T	p.P3118S	0.18	-	-
14	*IGSF10*	NM_178822	c.4869G>C	p.K1623N	0.2	-	-
14	*IL1RN*	NM_000577	c.403G>A	p.D135N	0.19	-	-
14	*KDM7A*	NM_030647	c.379C>T	p.R127C	0.19	-	COSM2861153
14	*KIAA0391*	NM_014672	c.1072A>G	p.I358V	0.25	-	-
14	*KNG1*	NM_000893	c.181G>A	p.E61K	0.24	-	-
14	*KSR2*	NM_173598	c.1402A>G	p.T468A	0.23	-	-
14	*LRCH2*	NM_020871	c.1522G>C	p.E508Q	0.34	-	-
14	*MACF1*	NM_012090	c.12435G>C	p.E4145D	0.22	-	-
14	*MED24*	NM_014815	c.700G>C	p.E234Q	0.17	-	COSM1479522
14	*MGA*	NM_001080541	c.2137G>C	p.D713H	0.33	-	-
14	*MRTO4*	NM_016183	c.131C>G	p.S44C	0.14	-	-
14	*MTR*	NM_000254	c.3632C>T	p.P1211L	0.14	-	-
14	*MUC12*	NM_001164462	c.5866C>A	p.P1956T	0.16	-	-
14	*NACA*	NM_001113202	c.10G>A	p.E4K	0.21	-	-
14	*NACC2*	NM_144653	c.1362G>T	p.K454N	0.15	-	-
14	*NADK*	NM_023018	c.1315G>A	p.E439K	0.22	-	-
14	*NEFH*	NM_021076	c.2836G>C	p.E946Q	0.31	-	-
14	*NLRP8*	NM_176811	c.676G>C	p.E226Q	0.18	-	-
14	*OPCML*	NM_002545	c.665-5C>T	(exon 5)	0.22	-	-
14	*PAGE1*	NM_003785	c.55T>A	p.S19T	0.4	-	-
14	*PCSK1*	NM_000439	c.1813C>T	p.R605C	0.25	-	-
14	*PIK3CA*	NM_006218	c.1633G>A	p.E545K	0.21	RCV000014631	COSM763
14	*PIK3CA*	NM_006218	c.3172A>C	p.I1058L	0.14	-	COSM479744
14	*PITPNC1*	NM_012417	c.856G>A	p.E286K	0.24	-	-
14	*PLXNA4*	NM_020911	c.5513G>A	p.R1838Q	0.12	-	-
14	*PTGFRN*	NM_020440	c.359C>T	p.S120L	0.19	-	-
14	*PTPRF*	NM_002840	c.2356G>A	p.G786S	0.32	-	-
14	*RNASE1*	NM_002933	c.112C>T	p.R38W	0.21	-	-

14	*RNF43*	NM_017763	c.476G>A	p.W159*	0.17	-	-
14	*SACS*	NM_014363	c.9002T>C	p.I3001T	0.33	-	-
14	*SCN2A*	NM_021007	c.3906C>G	p.I1302M	0.15	-	-
14	*SCRT1*	NM_031309	c.844G>A	p.G282S	0.25	-	-
14	*SERPINB3*	NM_006919	c.1084G>C	p.E362Q	0.22	-	-
14	*SLC4A7*	NM_003615	c.1010C>T	p.S337L	0.17	-	-
14	*SMARCB1*	NM_003073	c.744_745insCCCA	p.D251Hfs*31	0.37	-	-
14	*SMPD4*	NM_017751	c.803A>G	p.Y268C	0.17	-	-
14	*TEKT4*	NM_144705	c.325A>G	p.M109V	0.23	-	-
14	*UBE4A*	NM_004788	c.562-1G>C	(exon 6)	0.17	-	-
14	*UGT2B11*	NM_001073	c.1201G>C	p.D401H	0.13	-	-
14	*USP6NL*	NM_014688	c.1882C>T	p.H628Y	0.26	-	-
14	*WWP1*	NM_007013	c.1807G>C	p.E603Q	0.15	-	-
14	*ZC3H7A*	NM_014153	c.1144C>T	p.L382F	0.25	-	-
14	*ZNF236*	NM_007345	c.821G>A	p.R274Q	0.19	-	-
14	*ZNF429*	NM_001001415	c.1649C>G	p.S550*	0.17	-	-
14	*ZNF534*	NM_001143938	c.1135G>C	p.E379Q	0.21	-	-
14	*ZNF727*	NM_001159522	c.317G>C	p.C106S	0.15	-	-
15	*C8orf44*	NM_019607	c.188A>G	p.K63R	0.4	-	-
15	*DYNAP*	NM_173629	c.374T>C	p.M125T	0.31	-	-
16	*AHNAK*	NM_001620	c.5341G>A	p.V1781I	0.14	-	-
16	*C14orf159*	NM_024952	c.497T>C	p.M166T	0.14	-	-
16	*CCDC116*	NM_152612	c.409C>T	p.R137C	0.13	-	-
16	*CCDC40*	NM_001243342	c.2884G>A	p.G962R	0.08	-	-
16	*CDK20*	NM_001170640	c.581C>T	p.T194M	0.06	-	-
16	*CLPS*	NM_001252597	c.1A>G	p.M1V	0.36	-	-
16	*DPCR1*	NM_080870	c.2878A>G	p.K960E	0.06	-	-
16	*DUSP14*	NM_007026	c.487C>T	p.R163C	0.11	-	-
16	*GPR179*	NM_001004334	c.235C>T	p.R79C	0.17	-	-
16	*JARID2*	NM_004973	c.2416G>A	p.V806I	0.21	-	-
16	*LMTK3*	NM_001080434	c.2951_2952insG	p.E985Rfs*506	0.1	-	-
16	*LRR1*	NM_152329	c.986G>A	p.R329Q	0.26	-	-
16	*PCDHB4*	NM_018938	c.1417A>C	p.S473R	0.41	-	-
16	*PCNX*	NM_014982	c.5567G>A	p.R1856H	0.08	-	-

16	*PLXDC2*	NM_032812	c.1268C>T	p.T423I	0.31	-	-
16	*SLC6A1*	NM_003042	c.650C>T	p.T217M	0.17	-	-
16	*TBXA2R*	NM_201636	c.1091C>T	p.A364V	0.28	-	-
16	*TCEB3C*	NM_145653	c.1150G>A	p.A384T	0.04	-	-
16	*UNC13A*	NM_001080421	c.1483C>T	p.R495C	0.13	-	-
16	*ZNF285*	NM_152354	c.254C>G	p.T85S	0.2	-	-
17	*ADGRB3*	NM_001704	c.3713C>T	p.S1238L	0.12	-	-
17	*AGAP2*	NM_001122772	c.788G>A	p.R263Q	0.1	-	-
17	*ATP8B2*	NM_020452	c.2188G>C	p.E730Q	0.09	-	-
17	*BAHD1*	NM_014952	c.1021G>C	p.E341Q	0.09	-	-
17	*CAST*	NM_173060	c.1618-3C>T	(exon 23)	0.1	-	-
17	*CHGB*	NM_001819	c.745C>T	p.H249Y	0.15	-	-
17	*CTNND2*	NM_001332	c.2654G>A	p.R885Q	0.12	-	-
17	*CYGB*	NM_134268	c.164C>T	p.S55L	0.08	-	-
17	*DDX53*	NM_182699	c.1189G>A	p.V397M	0.08	-	-
17	*HOXA3*	NM_030661	c.732G>A	p.M244I	0.09	-	-
17	*IDI1*	NM_004508	c.670G>A	p.E224K	0.27	-	-
17	*IL6ST*	NM_002184	c.2245T>A	p.S749T	0.06	-	-
17	*IQSEC1*	NM_014869	c.796G>A	p.E266K	0.13	-	-
17	*MOV10L1*	NM_018995	c.3352G>A	p.D1118N	0.12	-	-
17	*MUC16*	NM_024690	c.33380C>G	p.P11127R	0.1	-	-
17	*OR4N5*	NM_001004724	c.329G>C	p.G110A	0.06	-	-
17	*OR5M10*	NM_001004741	c.512C>G	p.S171C	0.08	-	-
17	*PIK3CA*	NM_006218	c.1624G>C	p.E542Q	0.08	-	COSM17442
17	*PLCL1*	NM_006226	c.482C>T	p.S161F	0.07	-	-
17	*UBTD2*	NM_152277	c.374C>T	p.P125L	0.23	-	-
17	*ZNF611*	NM_030972	c.949T>A	p.C317S	0.13	-	-
17	*ZNF728*	NM_001267716	c.505G>A	p.G169R	0.11	-	-
18	*AQP7*	NM_001170	c.101T>A	p.V34E	0.09	-	-
18	*HLA-B*	NM_005514	c.121C>A	p.R41S	0.31	-	-
18	*KANK2*	NM_015493	c.530C>T	p.P177L	0.33	-	-
18	*MUC2*	NM_002457	c.2770A>C	p.M924L	0.22	-	-
18	*ROR1*	NM_005012	c.2788G>A	p.E930K	0.06	-	-
18	*ZNF195*	NM_001242841	c.338A>G	p.N113S	0.26	-	-
19	*CYP26B1*	NM_019885	c.1190G>A	p.R397Q	0.14	-	-
19	*FUT7*	NM_004479	c.335G>A	p.R112Q	0.29	-	-

19	*IGSF22*	NM_173588	c.3247G>A	p.E1083K	0.21	-	-
19	*JAG2*	NM_002226	c.3702C>A	p.Y1234*	0.16	-	-
19	*MYRF*	NM_013279	c.1645G>C	p.G549R	0.12	-	-
19	*PCDHA9*	NM_014005	c.1061C>T	p.T354M	0.12	-	-
19	*PIK3CA*	NM_006218	c.1637A>G	p.Q546R	0.12	RCV000038672	COSM12459
19	*SLCO1B3*	NM_019844	c.1510T>A	p.C504S	0.15	-	-
19	*SOGA1*	NM_080627	c.2237T>C	p.F746S	0.13	-	-
19	*TTN*	NM_003319	c.31156C>T	p.R10386C	0.16	-	-
19	*UQCRFS1*	NM_006003	c.397G>A	p.V133I	0.13	-	-
21	*KRTAP4-11*	NM_033059	c.249C>G	p.S83R	0.26	-	-
21	*NPIPB5*	NM_001135865	c.1549C>T	p.R517C	0.05	-	-
21	*SPDYE1*	NM_175064	c.791A>G	p.H264R	0.1	-	-
39	*ACOT2*	NM_006821	c.620G>C	p.R207P	0.23	-	-
39	*ADRB1*	NM_000684	c.790C>T	p.R264C	0.21	-	-
39	*ALDH3B2*	NM_000695	c.10G>A	p.E4K	0.2	-	-
39	*BCL11B*	NM_001282238	c.2078C>T	p.S693L	0.17	-	-
39	*BCL9*	NM_004326	c.2518dupG	p.L841Pfs*21	0.08	-	-
39	*C10orf71*	NM_001135196	c.832G>C	p.E278Q	0.09	-	-
39	*CACNA1E*	NM_001205294	c.5082+3G>A	(exon 36)	0.13	-	-
39	*CASP8AP2*	NM_001137667	c.1600G>C	p.D534H	0.46	-	-
39	*CCDC121*	NM_001142683	c.848T>C	p.I283T	0.14	-	-
39	*CHRNA10*	NM_001303034	c.445G>C	p.E149Q	0.12	-	-
39	*CHRNA7*	NM_000746	c.1066G>A	p.A356T	0.05	-	-
39	*CLDN18*	NM_016369	c.139G>A	p.E47K	0.19	-	-
39	*COQ4*	NM_016035	c.567G>C	p.Q189H	0.25	-	-
39	*DHX35*	NM_001190809	c.1963C>T	p.Q655X	0.28	-	-
39	*DLGAP1*	NM_001003809	c.811G>A	p.G271R	0.24	-	-
39	*EPB41L3*	NM_001281533	c.475G>A	p.E159K	0.48	-	-
39	*FAM160A1*	NM_001109977	c.1621G>A	p.E541K	0.17	-	-
39	*FASN*	NM_004104	c.6060_6062delCTC	p.2020delS	0.12	-	-

39	*FCRL1*	NM_001159397	c.733G>T	p.E245X	0.17	-	-
39	*GAREM1*	NM_001242409	c.1402C>T	p.L468F	0.23	-	-
39	*GON4L*	NM_001282856	c.4960C>T	p.Q1654X	0.18	-	-
39	*HAUS4*	NM_001166269	c.196C>T	p.Q66X	0.16	-	-
39	*HPCAL4*	NM_001282396	c.163-5C>-	(exon 4)	0.32	-	-
39	*HTRA1*	NM_002775	c.1297G>A	p.V433I	0.21	-	-
39	*KIAA0754*	NM_015038	c.4118dupT	p.M1374Hfs*35	0.16	-	-
39	*KIAA1549*	NM_001164665	c.2179C>T	p.L727F	0.27	-	-
39	*KIAA1958*	NM_133465	c.1513T>G	p.S505A	0.1	-	-
39	*LILRA2*	NM_001290270	c.1166C>G	p.S389C	0.07	-	-
39	*LONP1*	NM_004793	c.1686-1G>A	(exon 11)	0.24	-	-
39	*METTL10*	NM_212554	c.8C>G	p.S3W	0.15	-	-
39	*MRM1*	NM_024864	c.80G>A	p.G27E	0.27	-	-
39	*MTCL1*	NM_015210	c.3328G>A	p.E1110K	0.09	-	-
39	*MYO10*	NM_012334	c.3079G>A	p.D1027N	0.08	-	-
39	*MYO7B*	NM_001080527	c.6037G>A	p.E2013K	0.18	-	-
39	*NDUFS8*	NM_002496	c.412C>T	p.R138C	0.07	-	-
39	*NEUROD1*	NM_002500	c.223G>A	p.E75K	0.2	-	-
39	*NOC2L*	NM_015658	c.2221G>A	p.E741K	0.42	-	-
39	*OR2T8*	NM_001005522	c.81G>T	p.M27I	0.26	-	-
39	*OVGP1*	NM_002557	c.1555C>T	p.P519S	0.18	-	-
39	*PCDHA9*	NM_014005	c.544G>A	p.V182M	0.2	-	-
39	*PEG3*	NM_001146186	c.4486C>G	p.Q1496E	0.17	-	-
39	*PLPP6*	NM_203453	c.611C>T	p.S204L	0.14	-	-
39	*PMFBP1*	NM_031293	c.1213G>A	p.E405K	0.29	-	-
39	*POM121L12*	NM_182595	c.308C>T	p.P103L	0.11	-	-
39	*PRDM13*	NM_021620	c.707C>T	p.S236L	0.26	-	-
39	*PTPRT*	NM_007050	c.886G>A	p.E296K	0.18	-	-
39	*RNF213*	NM_001256071	c.13115T>G	p.L4372R	0.23	-	-
39	*RPS6KA2*	NM_021135	c.1901C>T	p.S634F	0.26	-	-
39	*RRBP1*	NM_004587	c.710T>G	p.L237R	0.13	-	-

39	*RRP7A*	NM_015703	c.712C>A	p.L238M	0.12	-	-
39	*RTL1*	NM_001134888	c.2972G>A	p.R991Q	0.08	-	-
39	*SALL3*	NM_171999	c.3718C>T	p.Q1240X	0.12	-	-
39	*SETD7*	NM_001306199	c.264C>G	p.D88E	0.12	-	-
39	*SHISA7*	NM_001145176	c.976+3G>A	(exon 3)	0.22	-	-
39	*SOX12*	NM_006943	c.856G>A	p.E286K	0.16	-	-
39	*SPATA31D1*	NM_001001670	c.1890G>T	p.Q630H	0.21	-	-
39	*TBX4*	NM_018488	c.1055G>A	p.R352Q	0.22	-	-
39	*THEMIS2*	NM_001039477	c.160C>T	p.Q54X	0.18	-	-
39	*TNKS1BP1*	NM_033396	c.3718G>A	p.E1240K	0.09	-	-
39	*TNR*	NM_003285	c.3219G>C	p.L1073F	0.3	-	-
39	*TP53BP1*	NM_001141979	c.5764G>A	p.V1922M	0.21	-	-
39	*TUBB4B*	NM_006088	c.1234G>A	p.E412K	0.19	-	-
39	*TULP4*	NM_001007466	c.1972T>A	p.F658I	0.24	-	-
39	*USP40*	NM_018218	c.488G>A	p.G163E	0.23	-	-
39	*ZNF711*	NM_021998	c.1442G>C	p.C481S	0.57	-	-
39	*ZNRF3*	NM_001206998	c.2221G>A	p.E741K	0.13	-	-
39	*ZSCAN10*	NM_001282415	c.925G>A	p.A309T	0.22	-	-
40	*ABCA1*	NM_005502	c.2140G>A	p.D714N	0.15	-	-
40	*ADH1A*	NM_000667	c.944G>A	p.W315X	0.08	-	-
40	*AMIGO2*	NM_181847	c.174C>A	p.N58K	0.16	-	-
40	*BTD*	NM_000060	c.809C>T	p.T270I	0.12	-	-
40	*C19orf24*	NM_017914	c.80A>G	p.E27G	0.17	-	-
40	*C2orf69*	NM_153689	c.1012G>A	p.D338N	0.13	-	-
40	*CAPN7*	NM_014296	c.1810G>A	p.E604K	0.34	-	-
40	*CARNS1*	NM_020811	c.214G>A	p.D72N	0.16	-	-
40	*CDCA5*	NM_080668	c.404A>G	p.D135G	0.18	-	-
40	*CENPF*	NM_016343	c.3115C>G	p.L1039V	0.3	-	-
40	*CTTNBP2*	NM_033427	c.3201C>G	p.S1067R	0.23	-	-
40	*CUX1*	NM_001202543	c.2893G>T	p.E965X	0.21	-	-
40	*CYFIP1*	NM_014608	c.44A>G	p.D15G	0.15	-	-

40	*DDX54*	NM_001111322	c.1505C>T	p.S502L	0.17	-	-
40	*DFNB31*	NM_001173425	c.334G>A	p.E112K	0.17	-	-
40	*DPEP3*	NM_001129758	c.1414C>T	p.H472Y	0.14	-	-
40	*DPEP3*	NM_001129758	c.590C>G	p.S197C	0.17	-	-
40	*EP300*	NM_001429	c.3798G>A	p.W1266X	0.37	-	-
40	*EPHB2*	NM_001309192	c.1237G>A	p.G413S	0.16	-	-
40	*EPHB3*	NM_004443	c.514_515insGCCGT	p.V174Afs*175	0.09	-	-
40	*ERBB3*	NM_001982	c.2773G>A	p.E925K	0.12	-	-
40	*FAM179B*	NM_001308120	c.280G>A	p.E94K	0.16	-	-
40	*FCHO1*	NM_001161359	c.1189G>A	p.E397K	0.35	-	-
40	*FSIP2*	NM_173651	c.16366G>A	p.D5456N	0.15	-	-
40	*GNPAT*	NM_001316350	c.35C>T	p.S12F	0.1	-	-
40	*GPC2*	NM_152742	c.1487-1G>A	(exon 10)	0.19	-	-
40	*GPRASP2*	NM_001184875	c.1795G>A	p.E599K	0.21	-	-
40	*GSX2*	NM_133267	c.791C>T	p.T264M	0.15	-	-
40	*HELZ2*	NM_033405	c.3260A>T	p.Q1087L	0.07	-	-
40	*HIST1H1T*	NM_005323	c.148G>A	p.E50K	0.12	-	-
40	*HMX2*	NM_005519	c.707C>T	p.S236L	0.06	-	-
40	*HTR6*	NM_000871	c.1207G>A	p.E403K	0.2	-	-
40	*KDM3B*	NM_016604	c.4789G>A	p.E1597K	0.18	-	-
40	*KIR2DL1*	NM_014218	c.854G>A	p.R285K	0.11	-	-
40	*LENG1*	NM_024316	c.130G>A	p.E44K	0.24	-	-
40	*LMAN1*	NM_005570	c.304G>A	p.E102K	0.18	-	-
40	*LTBR*	NM_001270987	c.1111G>A	p.E371K	0.13	-	-
40	*LTN1*	NM_015565	c.1417G>A	p.E473K	0.23	-	-
40	*LTN1*	NM_015565	c.1426G>T	p.E476X	0.22	-	-
40	*MAGEB17*	NM_001277307	c.730G>A	p.E244K	0.14	-	-
40	*MC5R*	NM_005913	c.788C>G	p.S263C	0.18	-	-
40	*MLLT4*	NM_001291964	c.4996C>T	p.Q1666X	0.16	-	-

40	*MTMR1*	NM_001306145	c.961G>A	p.E321K	0.22	-	-
40	*MUC4*	NM_018406	c.4775C>T	p.S1592L	0.05	-	-
40	*NEURL1*	NM_004210	c.790G>A	p.E264K	0.13	-	-
40	*NOP56*	NM_006392	c.1256A>T	p.D419V	0.06	-	-
40	*NPIPB5*	NM_001135865	c.1861C>T	p.Q621X	0.08	-	-
40	*NXF5*	NM_032946	c.42C>G	p.F14L	0.2	-	-
40	*OVOL1*	NM_004561	c.151G>A	p.V51M	0.12	-	-
40	*PARVG*	NM_001137605	c.616C>G	p.P206A	0.15	-	-
40	*PCLO*	NM_014510	c.6511G>A	p.E2171K	0.21	-	-
40	*PDE12*	NM_177966	c.998A>G	p.N333S	0.24	-	-
40	*PDE4DIP*	NM_001002811	c.101C>T	p.S34L	0.07	-	-
40	*PELI3*	NM_001243136	c.163G>A	p.D55N	0.14	-	-
40	*PMFBP1*	NM_031293	c.1720C>G	p.Q574E	0.17	-	-
40	*POLD1*	NM_001308632	c.1682G>A	p.R561Q	0.16	-	-
40	*RDH11*	NM_001252650	c.407C>G	p.S136W	0.16	-	-
40	*RNF123*	NM_022064	c.922C>T	p.R308W	0.21	-	-
40	*RREB1*	NM_001003698	c.364G>A	p.E122K	0.2	-	-
40	*SIK2*	NM_015191	c.2125G>C	p.E709Q	0.15	-	-
40	*SKIDA1*	NM_207371	c.1520A>C	p.D507A	0.22	-	-
40	*SLC9A7*	NM_001257291	c.714G>C	p.K238N	0.34	-	-
40	*SRCAP*	NM_006662	c.2518C>G	p.R840G	0.16	-	-
40	*SUFU*	NM_001178133	c.1099G>C	p.E367Q	0.18	-	-
40	*SUMF2*	NM_001042469	c.167G>A	p.R56K	0.18	-	-
40	*TINAGL1*	NM_001204414	c.112C>T	p.R38W	0.16	-	-
40	*TMEM63A*	NM_014698	c.127C>T	p.Q43X	0.31	-	-
40	*TRAPPC13*	NM_001093756	c.617C>T	p.S206L	0.36	-	-
40	*TTN*	NM_003319	c.75965C>G	p.S25322C	0.15	-	-
40	*TTN*	NM_003319	c.76951C>T	p.H25651Y	0.13	-	-
40	*TTN*	NM_003319	c.77291C>T	p.S25764L	0.23	-	-

40	*TTN*	NM_003319	c.78890C>G	p.S26297C	0.19	-	-
40	*UCP2*	NM_003355	c.334G>C	p.E112Q	0.17	-	-
40	*USP29*	NM_020903	c.1907G>C	p.R636T	0.22	-	-
40	*WDR24*	NM_032259	c.1362C>G	p.I454M	0.23	-	-
40	*WDR38*	NM_001276376	c.614C>T	p.S205F	0.16	-	-
40	*ZSWIM5*	NM_020883	c.3397C>T	p.R1133C	0.17	-	-
41	*ARPP21*	NM_001267617	c.2216C>T	p.P739L	0.1	-	-
41	*ERBB2*	NM_001289937	c.929C>T	p.S310F	0.11	-	COSM48358
42	*ADAMTS16*	NM_139056	c.2738C>T	p.T913I	0.22	-	-
42	*ADRA2C*	NM_000683	c.607G>A	p.G203S	0.07	-	-
42	*AGRP*	NM_001138	c.161C>T	p.T54I	0.23	-	-
42	*APOH*	NM_000042	c.460C>T	p.R154C	0.18	-	-
42	*ATXN2*	NM_001310123	c.2290C>T	p.Q764X	0.23	-	-
42	*CAD*	NM_001306079	c.6229G>A	p.D2077N	0.17	-	-
42	*CAD*	NM_001306079	c.6327G>A	p.M2109I	0.21	-	-
42	*CDKN2A*	NM_000077	c.245T>G	p.V82G	0.21	-	-
42	*CEACAM5*	NM_001291484	c.546T>A	p.N182K	0.2	-	-
42	*CEP295*	NM_033395	c.5978A>G	p.K1993R	0.27	-	-
42	*CHRNA4*	NM_000744	c.1034G>A	p.R345H	0.12	-	-
42	*COL22A1*	NM_152888	c.359C>T	p.A120V	0.11	-	-
42	*DDX53*	NM_182699	c.784C>G	p.L262V	0.43	-	-
42	*DOT1L*	NM_032482	c.448G>C	p.E150Q	0.15	-	-
42	*FARP1*	NM_005766	c.2527G>A	p.E843K	0.08	-	-
42	*GTF3C4*	NM_012204	c.523G>A	p.D175N	0.29	-	-
42	*GYPC*	NM_016815	c.86G>C	p.R29T	0.16	-	-
42	*HHIPL2*	NM_024746	c.562G>A	p.D188N	0.12	-	-
42	*HSF1*	NM_005526	c.718T>G	p.Y240D	0.13	-	-
42	*KIR3DL2*	NM_001242867	c.486G>T	p.E162D	0.05	-	-
42	*KLK4*	NM_001302961	c.223G>A	p.V75M	0.06	-	-
42	*LIPE*	NM_005357	c.3049C>A	p.R1017S	0.25	-	-
42	*LRRN2*	NM_201630	c.1721G>A	p.R574H	0.13	-	-
42	*MAD1L1*	NM_001304525	c.349G>A	p.D117N	0.12	-	-

42	*OR51S1*	NM_001004758	c.137C>G	p.S46C	0.21	-	-
42	*OR6F1*	NM_001005286	c.422C>T	p.S141L	0.25	-	-
42	*PLK1*	NM_005030	c.1711G>A	p.G571S	0.06	-	-
42	*PPP1R26*	NM_014811	c.1834G>A	p.E612K	0.27	-	-
42	*RAB40C*	NM_021168	c.552G>A	p.W184X	0.09	-	-
42	*SH2D6*	NM_198482	c.490C>G	p.R164G	0.1	-	-
42	*SLC18A3*	NM_003055	c.358G>A	p.E120K	0.17	-	-
42	*SMAD3*	NM_001145104	c.654G>T	p.Q218H	0.14	-	-
42	*SMYD4*	NM_052928	c.892C>T	p.R298X	0.14	-	-
42	*SNAP25*	NM_003081	c.531G>C	p.Q177H	0.37	-	-
42	*SORCS3*	NM_014978	c.3202G>A	p.E1068K	0.21	-	-
42	*SPEF1*	NM_015417	c.553C>A	p.Q185K	0.1	-	-
42	*SRC*	NM_005417	c.142C>T	p.R48C	0.2	-	-
42	*TP63*	NM_001114980	c.1543G>A	p.E515K	0.18	-	-
42	*WNT7A*	NM_004625	c.1032G>C	p.E344D	0.12	-	-
43	*IRS2*	NM_003749	c.3751C>G	p.L1251V	0.07	-	-
43	*KRTAP3-3*	NM_033185	c.20G>A	p.R7Q	0.22	-	-
43	*MUC5B*	NM_002458	c.16800+2T>A	(exon 45)	0.06	-	-
43	*PCDHA2*	NM_018905	c.2252C>T	p.S751L	0.07	-	-
43	*TCOF1*	NM_000356	c.959C>T	p.A320V	0.14	-	-
44	*C19orf66*	NM_001308277	c.301G>A	p.D101N	0.06	-	-
44	*CYP2A6*	NM_000762	c.932G>A	p.R311H	0.06	-	-
44	*ENGASE*	NM_001042573	c.1185C>G	p.I395M	0.08	-	-
44	*FAM184B*	NM_015688	c.579G>A	p.M193I	0.11	-	-
44	*FARS2*	NM_006567	c.90G>T	p.Q30H	0.06	-	-
44	*FBN3*	NM_032447	c.8228G>A	p.R2743H	0.13	-	-
44	*FUT3*	NM_001097641	c.179_187delGCCCCACCC	p.58_60delPTR	0.21	-	-
44	*LOC389895*	NM_001271560	c.451G>T	p.A151S	0.11	-	-
44	*LRRC40*	NM_017768	c.131C>A	p.S44X	0.12	-	-
44	*MAP3K10*	NM_002446	c.1403G>A	p.R468Q	0.08	-	-
44	*MYO15A*	NM_016239	c.1375G>A	p.E459K	0.07	-	-
44	*PCDH8*	NM_002590	c.3110G>A	p.R1037H	0.12	-	-
44	*PLEC*	NM_201378	c.8846T>G	p.V2949G	0.05	-	-

44	*RHOBTB2*	NM_001160037	c.230G>A	p.R77Q	0.1	-	-
44	*RLIM*	NM_016120	c.176G>A	p.S59N	0.14	-	-
44	*SIGLEC12*	NM_033329	c.553A>G	p.T185A	0.13	-	-
44	*TP53*	NM_000546	c.827_830delCCTG	p.A276Vfs*68	0.11	-	-
44	*UBQLN3*	NM_017481	c.1400C>T	p.A467V	0.13	-	-
44	*ZNF423*	NM_001271620	c.1805A>G	p.Q602R	0.06	-	-
45	*DACT1*	NM_001079520	c.2009C>T	p.A670V	0.1	-	-
45	*DUSP9*	NM_001395	c.527C>A	p.S176Y	0.32	-	-
45	*ICT1*	NM_001303265	c.158A>C	p.Q53P	0.07	-	-
45	*ITPR3*	NM_002224	c.1954A>C	p.I652L	0.05	-	-
45	*KCNJ4*	NM_004981	c.100A>C	p.N34H	0.05	-	-
45	*KCNK12*	NM_022055	c.862G>T	p.V288L	0.06	-	-
45	*KLHL26*	NM_018316	c.280G>A	p.G94S	0.09	-	-
45	*LILRA5*	NM_181985	c.461T>G	p.L154R	0.12	-	-
45	*MFSD12*	NM_001287529	c.1196T>G	p.M399R	0.07	-	-
45	*MYO9B*	NM_001130065	c.499A>C	p.K167Q	0.05	-	-
45	*PARP9*	NM_001146102	c.440G>A	p.G147E	0.14	-	-
45	*PCDHB3*	NM_018937	c.1708G>A	p.A570T	0.08	-	-
45	*PLXNB2*	NM_012401	c.4127_4135delTCCTGGAGC	p.1374_1376delLEL	0.11	-	-
45	*PRDM16*	NM_022114	c.2555C>T	p.P852L	0.14	-	-
45	*SCN5A*	NM_000335	c.2182G>A	p.V728I	0.09	-	-
45	*SNX32*	NM_152760	c.1057G>T	p.D353Y	0.09	-	-
45	*ZNF646*	NM_014699	c.1513A>C	p.M505L	0.07	-	-
46	*ADCK2*	NM_052853	c.175G>A	p.E59K	0.08	-	-
47	*ALPK2*	NM_052947	c.3524C>T	p.S1175L	0.18	-	-
47	*AP3B2*	NM_004644	c.3099-3C>T	(exon 26)	0.29	-	-
47	*APBA2*	NM_001130414	c.1646C>T	p.S549L	0.23	-	-
47	*BEST4*	NM_153274	c.130G>T	p.A44S	0.29	-	-
47	*C9orf64*	NM_032307	c.157C>T	p.H53Y	0.25	-	-
47	*CNOT1*	NM_001265612	c.4291C>T	p.R1431X	0.27	-	-
47	*COQ4*	NM_001305942	c.31C>G	p.R11G	0.24	-	-

47	*CTNND2*	NM_001332	c.65C>T	p.S22L	0.42	-	-
47	*DAPK1*	NM_001288729	c.2641C>T	p.Q881X	0.32	-	-
47	*DCAF8*	NM_015726	c.1153G>A	p.E385K	0.23	-	-
47	*DDOST*	NM_005216	c.870A>C	p.E290D	0.13	-	-
47	*DDX49*	NM_019070	c.1024G>A	p.A342T	0.31	-	-
47	*EHMT2*	NM_001289413	c.1820G>A	p.R607Q	0.44	-	-
47	*FCRL1*	NM_001159397	c.666G>C	p.E222D	0.32	-	-
47	*GSG1*	NM_001080554	c.241G>A	p.D81N	0.12	-	-
47	*HELZ*	NM_014877	c.4696G>A	p.E1566K	0.16	-	-
47	*IFT140*	NM_014714	c.2856G>A	p.M952I	0.25	-	-
47	*IFT140*	NM_014714	c.3430G>A	p.E1144K	0.27	-	-
47	*INSRR*	NM_014215	c.1504C>T	p.R502C	0.08	-	-
47	*ITGAL*	NM_001114380	c.2434G>A	p.A812T	0.29	-	-
47	*ITPKA*	NM_002220	c.1273G>A	p.D425N	0.18	-	-
47	*KIF25*	NM_030615	c.898G>A	p.A300T	0.46	-	-
47	*KRT38*	NM_006771	c.1121C>T	p.S374F	0.21	-	-
47	*LOC441155*	NM_001271675	c.433G>A	p.E145K	0.29	-	-
47	*LRP1*	NM_002332	c.2608C>G	p.R870G	0.1	-	-
47	*MAGI1*	NM_001033057	c.2087C>T	p.A696V	0.17	-	-
47	*METTL14*	NM_020961	c.1343_1344delCA	p.H449Rfs*71	0.25	-	-
47	*MICB*	NM_001289160	c.426G>A	p.M142I	0.36	-	-
47	*MYH9*	NM_002473	c.3488C>T	p.S1163L	0.21	-	-
47	*NEU2*	NM_005383	c.277G>A	p.A93T	0.19	-	-
47	*NLRP7*	NM_001127255	c.331G>T	p.D111Y	0.22	-	-
47	*ODF1*	NM_024410	c.520G>A	p.E174K	0.07	-	-
47	*PDE4A*	NM_001243121	c.46G>T	p.E16X	0.27	-	-
47	*PTH*	NM_000315	c.236C>T	p.S79F	0.25	-	-
47	*RBM8A*	NM_005105	c.40G>A	p.D14N	0.16	-	-
47	*RPS12*	NM_001016	c.7G>A	p.E3K	0.33	-	-
47	*SEC62*	NM_003262	c.1156G>A	p.E386K	0.22	-	-
47	*SELE*	NM_000450	c.820G>A	p.E274K	0.2	-	-

47	*SEMA4A*	NM_001193302	c.1027G>A	p.A343T	0.19	-	-
47	*SFI1*	NM_001258326	c.982C>T	p.H328Y	0.31	-	-
47	*SLC26A9*	NM_052934	c.1634T>A	p.I545N	0.23	-	-
47	*SORCS1*	NM_052918	c.2058+1G>A	(exon 15)	0.25	-	-
47	*TMEM132E*	NM_001304438	c.50C>T	p.S17L	0.29	-	-
47	*TP53*	NM_001126115	c.128G>A	p.R43H	0.4	RCV000161065	COSM3355994
47	*TPM2*	NM_003289	c.773-1->C	(exon 9)	0.18	-	-
47	*TUBGCP6*	NM_020461	c.3463C>T	p.R1155W	0.09	-	-
47	*ZIC3*	NM_003413	c.68G>A	p.R23H	0.08	-	-
47	*ZNF208*	NM_007153	c.205G>A	p.E69K	0.22	-	-
47	*ZNF787*	NM_001002836	c.508C>T	p.R170C	0.4	-	-
47	*ZNF831*	NM_178457	c.1100C>T	p.S367L	0.13	-	-
48	*ICT1*	NM_001303265	c.158A>C	p.Q53P	0.07	-	-

Table S5. Summary of possible driver mutations identified in this study

UPN	Gene	Reference	Nucleic acid change	Amino acid change	VAF	ClinVar	COSMIC
1	*GAS6-FOXA1*	-	-	(fusion gene)	0.10	-	-
2	*FOXA1*	NM_001310135	g.38064406G>A	(promoter)	0.21	-	-
2	*HIST1H2BC*	NM_003526	c.72G>C	p.K24N	0.20	-	-
2	*PIK3CA*	NM_006218	c.241G>A	p.E81K	0.28	-	COSM271871
3	*FOXA1*	NM_001310135	g.38064406G>A	(promoter)	0.08	-	-
3	*PIK3CA*	NM_006218	c.2176G>A	p.E726K	0.20	VCV000376476	COSM446019
5	*PIK3CA*	NM_006218	c.3019G>C	p.G1007R	0.09	-	COSM17443
6	*FOXA1*	NM_001310135	g.38064406G>T	(promoter)	0.01	-	-
8	*FOXA1*	NM_001310135	g.38064406G>A	(promoter)	0.01	-	-
14	*FOXA1*	NM_001310135	g.38064406G>A	(promoter)	0.01	-	-
14	*HIST1H2BB*	NM_021062	c.72G>C	p.K24N	0.19	-	COSM1169071
14	*PIK3CA*	NM_006218	c.1633G>A	p.E545K	0.21	RCV000014631	COSM763
14	*PIK3CA*	NM_006218	c.3172A>C	p.I1058L	0.15	-	COSM479744
14	*SMARCB1*	NM_003073	c.744_745insCCCA	p.D251Hfs*31	0.37	-	-
17	*PIK3CA*	NM_006218	c.1624G>C	p.E542Q	0.08	-	COSM17442
18	*FOXA1*	NM_001310135	g.38064406G>A	(promoter)	0.29	-	-
19	*PIK3CA*	NM_006218	c.1637A>G	p.Q546R	0.13	RCV000038672	COSM12459
25	*FOXA1*	NM_001310135	g.38064406G>A	(promoter)	0.03	-	-
35	*FOXA1*	NM_001310135	g.38064406G>A	(promoter)	0.05	-	-
40	*CUX1*	NM_001202543	c.2893G>T	p.E965X	0.21	-	-
40	*EP300*	NM_001429	c.3798G>A	p.W1266X	0.37	-	-
41	*ERBB2*	NM_001289937	c.929C>T	p.S310F	0.11	-	COSM48358
44	*TP53*	NM_000546	c.827_830delCCTG	p.A276Vfs*68	0.11	-	-
46	*FOXA1*	NM_001310135	g.38064406G>A	(promoter)	0.04	-	-
47	*TP53*	NM_001126115	c.128G>A	p.R43H	0.4	RCV000161065	COSM3355994
48	*FOXA1*	NM_001310135	g.38064406G>A	(promoter)	0.01	-	-

Table S6. Top 200 differentially expressed genes

Gene	baseMean	Log2 fold change	adjusted p-value	*FOXA1* target	*FOXA1* pathway	Keratin family
MLPH	*6291.8*	3.59	3.2E-147			
TSPAN1	*8694.4*	6.38	1.2E-75			
TMPRSS11D	*2394.2*	7.92	5.6E-68			
ANO7	*2972.0*	7.61	5.2E-65			
KRT2	*152699.4*	-12.53	1.0E-56			Y
GPX2	*2051.1*	3.42	5.3E-55			
MEF2A	*3745.1*	-1.98	4.4E-53			
PRR15	*1815.5*	6.53	1.0E-52			
ABCC11	*2896.6*	8.88	1.3E-51			
SOS2	*2029.9*	-1.34	4.3E-51			
SEC14L2	*3122.7*	3.46	4.5E-51			
HHIPL2	*647.6*	7.81	9.3E-49			
FGFR4	*4917.3*	6.33	8.7E-47			
RHCG	*7665.3*	5.16	1.8E-46			
KMO	*288.8*	5.44	9.5E-46			
MUC5AC	*9131.8*	14.97	2.2E-44			
STXBP5	*2264.0*	-1.57	1.0E-43			
GAL3ST2	*349.4*	7.11	5.3E-42			
TMEM132A	*8050.6*	4.67	7.6E-42			
SPDEF	*6669.8*	4.75	1.1E-40	Y		
JAK2	*1150.8*	-1.87	3.0E-40			
PGD	*4916.6*	1.91	6.0E-40			
NETO2	*717.7*	4.19	1.8E-39			
VSIG2	*1890.2*	5.64	3.9E-39	Y		
CAPN9	*1374.6*	8.93	5.1E-39			
FUT3	*1316.4*	4.62	9.8E-39			
NRK	*504.4*	4.96	1.0E-37			
PTHLH	*3723.3*	7.46	2.7E-37			
ANKRD12	*4283.4*	-1.77	1.5E-36			
SYT8	*2227.0*	-5.27	1.6E-35			
SYNE4	*291.5*	3.09	1.9E-35			
FOXA3	*1377.8*	10.44	2.0E-35		Y	
LRP4	*3358.7*	-3.41	3.8E-35			
BAALC	*1241.3*	4.15	6.7E-35			
PGAP1	*1509.0*	-2.22	1.2E-34			
SEPT10	*2239.2*	-1.28	4.8E-34			
YIF1B	*1907.5*	1.33	1.1E-33			
TFF1	*3927.8*	15.44	2.1E-33		Y	

CAPN8	*961.7*	5.15	6.1E-33			
SLC44A4	*3451.5*	5.08	1.2E-32			
SPRR1A	*26589.6*	5.28	5.1E-32			
ZNF593	*1131.7*	1.82	7.6E-32			
PDK3	*2532.0*	-2.49	8.1E-32			
KRT6A	*88088.3*	5.13	1.0E-31			Y
GJB2	*45826.9*	3.76	3.1E-31			
KRT8	*14812.9*	4.20	7.1E-31			Y
KIAA1328	*228.7*	-1.58	1.1E-30			
CNDP2	*7070.2*	1.47	1.3E-30			
ERP27	*489.3*	3.04	3.0E-30			
LOC101929613	*465.0*	8.20	3.3E-30			
HBA1	*175.5*	-5.15	4.2E-30			
ANTXR1	*4727.9*	-2.74	7.7E-30			
ARRDC1	*4439.7*	2.09	4.2E-29			
GSTP1	*37241.3*	2.13	6.9E-29			
DST	*24044.7*	-1.69	7.0E-29			
TBC1D30	*376.9*	3.14	8.6E-29			
STRA6	*1211.6*	7.93	8.8E-29			
KRBA1	*1154.2*	1.52	1.0E-28			
LRRC31	*305.9*	8.21	1.0E-28			
ABCG4	*654.2*	5.68	7.5E-28			
PNMT	*926.4*	6.58	1.4E-27			
ASL	*1303.0*	1.52	1.8E-27			
CDKL1	*202.4*	-1.60	3.7E-27			
KIAA2026	*1909.1*	-1.15	4.0E-27			
AGR2	*7079.3*	6.04	4.3E-27			
RGMB	*1959.2*	-2.10	4.3E-27			
HDAC1	*4293.1*	1.10	5.0E-27			
CDON	*2126.9*	-3.22	5.2E-27			
CCDC103	*97.3*	2.54	5.2E-27			
EPHB1	*241.5*	-4.64	5.2E-27			
SPRY1	*1088.7*	-2.39	5.2E-27	Y		
QKI	*3722.9*	-1.59	6.3E-27			
FOXA1	*3816.5*	4.22	9.7E-27		Y	
COA3	*2114.4*	1.26	1.0E-26			
PCGF5	*1751.2*	-2.34	1.0E-26			
AKR7A3	*397.6*	4.39	2.2E-26			
CXCL17	*334.3*	6.60	2.3E-26			
POLD4	*3714.0*	1.83	2.9E-26	Y		
MBNL1	*5910.3*	-1.26	3.0E-26			
VSIG1	*959.5*	5.51	3.5E-26			

GNB4	*4613.4*	-2.42	3.7E-26		
B4GALNT2	*2949.3*	6.99	4.3E-26		
CEACAM6	*20151.5*	4.52	7.0E-26		
AEN	*1637.2*	1.72	2.1E-25		
ISG20	*1457.6*	3.34	2.5E-25		
FOXP2	*344.8*	-3.21	2.7E-25	Y	
EFHD1	*3316.0*	3.84	2.9E-25		
MAT1A	*115.4*	-5.73	3.4E-25		
GBP6	*2157.5*	4.46	4.0E-25		
ST6GALNAC1	*355.0*	3.40	5.4E-25		
MBOAT7	*3696.0*	1.23	5.5E-25		
AP1M2	*3390.2*	1.42	5.7E-25		
CYP2S1	*929.3*	2.00	5.9E-25		
TNNI2	*431.1*	-7.06	9.2E-25		
PJA2	*4666.1*	-1.12	1.2E-24		
SMIM22	*857.8*	4.15	1.7E-24		
MUC7	*243.3*	-11.56	1.7E-24		
SOCS5	*1092.9*	-1.04	2.0E-24		
KRT19	*47366.4*	4.40	2.2E-24		Y
FAM122A	*973.4*	-0.81	2.3E-24	Y	
CLN3	*2730.3*	1.30	2.3E-24		
TIMM8B	*1504.4*	1.66	4.7E-24		
SWI5	*525.3*	1.12	5.0E-24		
KYNU	*1437.2*	4.38	5.4E-24		
LDLRAD1	*166.0*	6.12	6.3E-24		
CYB561D2	*756.9*	1.58	9.6E-24		
MIR31HG	*105.8*	6.81	1.1E-23		
CAV2	*2831.8*	-1.84	1.2E-23		
CTDSPL	*3717.1*	-1.65	1.9E-23		
JADE1	*993.4*	-2.11	2.4E-23	Y	
TUFM	*8269.6*	1.17	2.4E-23		
KLK13	*6683.4*	4.52	3.2E-23		
NR3C1	*3242.5*	-2.03	3.2E-23		
WDR47	*2324.4*	-1.20	3.5E-23		
FER	*1301.5*	-1.08	3.7E-23		
TRIM16	*2332.3*	1.80	5.0E-23		
BIK	*452.2*	2.80	5.2E-23		
LSM11	*307.4*	-1.39	5.4E-23		
ZNF654	*816.8*	-1.10	6.5E-23		
SNRNP35	*1345.2*	1.00	8.8E-23		
CSTA	*44252.8*	2.89	9.2E-23		
DDX39A	*2878.3*	1.36	9.3E-23		

TOX3	*730.9*	4.39	1.1E-22		
AFP	*625.6*	11.63	1.1E-22		
USP12	*1629.5*	-1.25	1.3E-22		
SNTB2	*1707.3*	-1.34	1.5E-22		
HSP90AB1	*20619.7*	0.79	2.4E-22		
NFIX	*11628.3*	-2.05	2.7E-22		
ABCC1	*5427.2*	1.50	4.3E-22		
FARSA	*3609.3*	1.07	4.3E-22		
LINC00514	*200.0*	7.46	4.6E-22		
SOX11	*651.4*	7.26	4.7E-22		
MKLN1	*2712.7*	-1.00	4.7E-22		
TFAP2B	*6897.3*	3.36	6.1E-22		
DENND4C	*3647.5*	-1.59	6.6E-22		
CXCL14	*28129.5*	-2.26	7.8E-22		
ETV6	*1958.7*	-1.34	7.8E-22		
HM13	*4588.3*	1.34	8.1E-22		
LPIN1	*775.7*	-1.91	8.1E-22		
HID1	*3461.8*	2.96	9.5E-22	Y	
B9D1	*421.0*	1.82	9.7E-22		
KRT18	*3956.5*	3.56	1.0E-21		Y
OGFRL1	*931.5*	-1.80	1.1E-21		
SLC9A3R1	*11639.6*	2.70	1.4E-21	Y	
NPW	*3371.2*	7.23	1.6E-21		
BCAS1	*1630.9*	3.72	1.8E-21	Y	
ALDOA	*36527.7*	1.26	1.9E-21		
HMGB3	*1136.6*	1.91	1.9E-21		
SMPDL3B	*468.2*	3.75	1.9E-21		
CDHR1	*12616.3*	-3.78	1.9E-21		
SCAND1	*2996.2*	1.40	1.9E-21		
DYNC1I1	*73.4*	-4.68	2.1E-21		
SERPINB3	*30391.2*	5.83	2.6E-21		
CNGB1	*116.0*	6.74	2.6E-21		
TNFRSF21	*2297.2*	1.73	2.8E-21		
TMEM208	*1784.5*	1.35	3.0E-21	Y	
RPL7	*457.1*	-1.87	3.1E-21		
MRPL12	*3375.2*	1.65	3.1E-21		
KRT17	*123592.3*	5.21	4.1E-21	Y	Y
CXCL6	*953.1*	11.33	4.5E-21		
FAM175A	*274.0*	-1.84	5.3E-21		
MANF	*3643.8*	1.67	5.7E-21		
JOSD2	*1264.5*	1.10	7.0E-21		
PRSS27	*2325.6*	4.17	7.0E-21		

FAM177B	*428.7*	8.54	7.7E-21		
SPRR1B	*56743.8*	4.15	8.4E-21		
ELK4	*2680.5*	-1.25	8.4E-21		
ZBTB33	*2119.2*	-1.48	9.5E-21		
SMAD5	*3665.5*	-1.20	9.7E-21	Y	
HMGCS2	*8114.4*	4.49	1.0E-20		
CCT3	*9891.2*	0.92	1.1E-20		
ARHGAP31	*1436.3*	-2.25	1.1E-20		
MAML2	*906.0*	-2.69	1.2E-20		
MRPS33	*1246.5*	1.16	1.2E-20		
RAB30	*707.9*	-2.06	1.3E-20		
SLC25A39	*6605.8*	1.28	1.3E-20		
GNG12	*9709.2*	-1.17	1.4E-20		
KRT16	*63038.5*	5.27	1.5E-20		Y
PYCR1	*2667.7*	3.32	1.6E-20		
SNRNP25	*903.0*	1.38	1.6E-20		
ATP13A1	*4315.1*	1.01	1.9E-20		
PXK	*615.3*	-1.68	3.0E-20		
HIST1H2BH	*38.9*	2.64	3.2E-20		
RSBN1	*914.0*	-1.36	3.4E-20		
C9orf116	*117.3*	2.93	3.7E-20		
H2AFX	*2146.6*	1.65	4.3E-20		
PRKAB2	*1397.2*	-1.54	4.7E-20		
ATP7B	*1375.0*	3.32	9.2E-20		
PAX9	*355.6*	4.57	9.8E-20		
CCDC167	*373.0*	1.75	1.2E-19		
LBX2	*55.7*	4.31	1.2E-19		
CAPN13	*515.0*	5.41	1.4E-19		
AKAP11	*2649.5*	-1.12	1.6E-19		
ERVMER34-1	*156.1*	3.04	1.7E-19		
SLC26A2	*1874.4*	-3.36	2.0E-19		
KPNA2	*2185.7*	2.25	2.0E-19		
DSTYK	*1977.5*	-1.30	2.4E-19		
ALAS1	*3561.0*	1.61	2.5E-19		
GABRP	*708.4*	-5.31	2.6E-19		
SMAGP	*1552.4*	1.48	2.8E-19		

Positive Log_2 fold change values indicate upregulation in patient samples. Y, yes
